# Laser Powder Bed Fusion of Molybdenum and Mo-0.1SiC Studied by Positron Annihilation Lifetime Spectroscopy and Electron Backscatter Diffraction Methods †

**DOI:** 10.3390/ma16041636

**Published:** 2023-02-16

**Authors:** Nathan E. Ellsworth, Joshua R. Machacek, Ryan A. Kemnitz, Cayla C. Eckley, Brianna M. Sexton, Joel A. Gearhart, Larry W. Burggraf

**Affiliations:** 1 Department of Engineering Physics, Air Force Institute of Technology, 2950 Hobson Way, Wright-Patterson AFB, OH 45433, USA; 2 Research School of Physics, Australian National University, Canberra 2601, Australia; 3 Department of Aeronautics and Astronautics, Air Force Institute of Technology, 2950 Hobson Way, Wright-Patterson AFB, OH 45433, USA

**Keywords:** additive manufacturing, laser powder bed fusion, selective laser melting, molybdenum, silicon carbide, nanoparticles, microstructure, positron annihilation lifetime spectroscopy

## Abstract

Positron annihilation lifetime spectroscopy (PALS) has been used for the first time to investigate the microstructure of additively manufactured molybdenum. Despite the wide applicability of positron annihilation spectroscopy techniques to the defect analysis of metals, they have only been used sparingly to monitor the microstructural evolution of additively manufactured metals. Molybdenum and molybdenum with a dilute addition (0.1 wt%) of nano-sized silicon carbide, prepared via laser powder bed fusion (LPBF) at four different scan speeds: 100, 200, 400, and 800 mm/s, were studied by PALS and compared with electron backscatter diffraction analysis. The aim of this study was to clarify the extent to which PALS can be used to identify microstructural changes resulting from varying LPBF process parameters. Grain sizes and misorientation results do not correlate with positron lifetimes indicating the positrons are sampling regions within the grains. Positron annihilation spectroscopy identified the presence of dislocations and nano-voids not revealed through electron microscopy techniques and correlated with the findings of SiO_2_ nanoparticles in the samples prepared with silicon carbide. The comparison of results indicates the usefulness of positron techniques to characterize nano-structure in additively manufactured metals due to the significant increase in atomic-level information.

## 1. Introduction

Positron annihilation spectroscopy (PAS) methods have been successfully used to investigate microstructural behavior during the heat treatments of defected and damaged metals since the method was first proposed in the 1960s and 1970s [1]. Most recently, Dryzek et al. studied defect evolution in the transition metals molybdenum, rhenium, vanadium, iron, and copper [2,3,4,5,6,7], under recovery and recrystallization conditions. Furthermore, correlating the positron information with the more traditional electron microscopy, X-ray, and microhardness techniques provided evidence that PAS techniques are needed for the study of refractory metals. Notably, the metals used were produced through standard manufacturing methods and deformed through relatively well-understood mechanical processes. Due to only a small number of available studies, it remains unclear to what level correlating PAS and electron microscopy techniques will prove useful for less understood mechanical and microstructural processes such as those occurring in laser powder bed fusion (LPBF).

In comparison to traditional manufacturing techniques, AM allows for the production of complex three-dimensional components in a manner that is more time, material, and cost-effective, but refractory metals, such as molybdenum, offer challenges due to their high melting points and ductile-to-brittle transition temperatures (DBTT) [8]. Oxygen impurities can increase the DBTT and the susceptibility of the consolidated material to cracking [9]. The microstructures of additively manufactured (AM) refractory metals lack the consistent, well-organized microstructures expected from standardized, traditional processing. Due to the rapid heating/cooling cycle involved in AM methods such as LPBF, temperature gradients exist that cause thermally-induced stresses to accumulate and result in cracking as the material cools [10]. Along with lack-of-fusion defects due to insufficient energy input, key-hole pores occur when there is enough energy input to destabilize the melt pool and trap gas bubbles upon its collapse [11]. These types of defects represent two of the largest classes of defects that are deleterious to the mechanical properties of AM metals and require detection during or after the manufacturing process to ensure parts are of sufficient quality.

Lack-of-fusion and key-hole pores are both micron-scale which allows the use of numerous methods to detect their formation and presence, both while undergoing consolidation and in the final state [12]. In addition to the standard destructive techniques such as hardness, compression, and tensile testing, as well as optical and electron microscopy, a large number of various non-destructive techniques have been applied to the AM process in order to characterize the consolidated material both in situ and post-build. High-speed cameras, pyrometers, and infrared (IR) cameras allow for monitoring of melt pool geometry, shape, and temperature distribution [13]. Macroscopic defects such as cracks, lack-of-fusion, and key-hole pores can be detected by IR cameras and X-ray imaging [14]. While these methods can identify micron-scale defects, their use in the bulk observation of smaller defects is limited.

Nanometer-sized defects and voids are more difficult to detect using conventional methods, especially over a relatively large area or volume of consolidated material or in a non-destructive manner. Voids in the nanometer range are a common defect in metals under deformation [15] where the void surfaces allow dislocations to nucleate and evolve [16]. Nano-void formation can occur as a result of the evolution of point defects to planar clusters and dislocation loops under deformation and thermal annealing after rapid quenching from high temperatures [17]. All of these microstructural processes occur during the LPBF process. Knowledge of nano-void formation and evolution is important due to their negative impact on macroscopic mechanical properties. Measurements using positrons have the advantage over the other techniques used in evaluating AM materials because they probe a three-dimensional subsurface volume that can be hundreds of microns deep instead of a small, select surface area found in electron and optical microscopy. Advancements in measurement and analysis have developed the ability of PAS techniques to identify single-atom vacancy-type defects [18] and can characterize microstructures at defect concentrations from 10${}^{-7}$ to 10${}^{-4}$ per atom [19]. They are highly sensitive to open-volume vacancy-type defects such as dislocations, monovacancies, divacancies, and clusters of vacancies at the atomic scale due to the lower electron density found here than in the bulk metal. The absence of a positively charged nuclear core leads to trapping at negatively charged defects. The type and size of the defect has an impact on the annihilation rate and characteristics which PAS experimental techniques can decipher. The lifetime of a positron within a void scales with the size of the vacancy-type defect up to a void size of around 100 nm [20], indicating the sensitivity of these techniques. While various experimental methods from atomic force microscopy and optical microscopy to X-ray and neutron scattering, offer analysis of defect sizes from hundreds of microns down to tens of nanometers, PAS techniques can measure defect sizes that are on the order of atomic radii. Transmission electron microscopy can observe dislocations and nano-scale defects; however, the preparation necessary far exceeds what is required for positron spectroscopy. Due to bulk, non-destructive, nano-sized defect characterization, we anticipate that positron spectroscopy has the potential for product monitoring during AM processing.

This work expands on the microscopy and mechanical testing of the LPBF of pure molybdenum and molybdenum with a dilute addition (0.1 wt%) of silicon carbide (Mo-0.1SiC) [21] by applying texture, grain boundary, and crystallographic orientation analysis through electron backscatter diffraction (EBSD) and microstructural analysis on the atomic-scale via PALS measurements. Literature reviews provide little evidence that PAS techniques have seen expansive use in characterizing AM materials [22,23]; however, their usefulness in identifying associated microstructural features should be expected. This work demonstrates that PALS, a subset of the broader techniques of PAS, can detect the presence of secondary-phase, silicon oxide nanoparticles and the evolution of nano-voids in LPBF molybdenum.

## 2. Materials and Methods

### 2.1. Specimen Preparation

The pure molybdenum and Mo-0.1SiC specimens used in this study were consolidated in the same manner as described in our last paper [21]. One cylindrical specimen, 15 mm in diameter and 5 mm in height, was produced for each laser scan speed of 100, 200, 400, and 800 mm/s. All cylindrical specimens were printed on copper substrates with a 0.5 mm offset at an initial scan speed of 800 mm/s as described in Eckley et al. [10] and Kemnitz et al. [8]. Wire electrical discharge machining was used to remove the specimens from the substrate and produce two identical cross-sectional surfaces, each with a thickness of roughly 2 mm, at each scan speed.

### 2.2. Electron Backscatter Diffraction Preparation

Samples were mounted in the transverse orientation in phenolic resin and the print surfaces were ground to a 1-micron surface finish. The samples were then vibratory polished for 17 h with colloidal silica to prepare them for electron backscatter diffraction (EBSD) analysis using an EDAX TEAM Pegasus system (Ametek Materials Analysis Division, Mahwah, NJ, USA). Three maps were obtained from each sample using a voltage of 18 kV and beam intensity of 18. Following their collection, EDAX orientation imaging microscopy (OIM) analysis software was used to process the maps and collect orientation, local misorientation, and texture data along with average grain size. A single iteration of neighbor orientation correlation clean-up was performed and pixels with a confidence interval (CI) of 0.2 and below were excluded.

### 2.3. Positron Measurements

The digital positron annihilation spectrometer used for this includes barium fluoride scintillators coupled to fast photomultiplier tubes (R3377) whose output was digitized using a 5GSs digitizer (CAEN VX1742, Viareggio, Italy). Its timing resolution is about 200 ps (FWHM).

The positron source was titanium encapsulated ^22^Na crystallites (A2305-2, POSN configuration, Eckert and Ziegler) with an activity of 10.1 μCi. The source is constructed so the ^22^Na is sealed between two, 5.08 μm thick, titanium foils and supported by two 0.25 mm thick titanium disks. The whole source is 19.1 mm in diameter while the active area has a 9.53 mm diameter [24].

The positrons from ^22^Na are emitted in a distribution of energies with a maximum and mean energy of 545 keV and 260 keV, respectively [25]. Due to the range of penetration depths, measurements are sensitive to the bulk and top printed layer which amounts to a bulk, near-surface method. The implantation profile for positrons emitted from a radioactive source can be empirically described by the equation,

(1) $$P\left(x\right)=e^{\left(-\alpha x\right)},$$

where α is the linear absorption coefficient [cm^−1^] and *x* is the depth [cm]. A number of empirical linear absorption coefficient expressions have been provided in the literature. Brandt and Paulin [26] developed the expression,

(2) $$\alpha =17\frac{\rho }{E_{max}^{1.49}},$$

where ρ is the density of the material [g/cm^3^] and *E_max_* is the maximum energy of the positron [MeV]. Mourino et al. [25,27], using experimental measurements involving materials with a wide range of atomic numbers, determined the following expression,

(3) $$\alpha =26.8Z^{0.15}\rho ,$$

where *Z* is the atomic number of the material under test. Dryzek and Singleton [28], through experimental and theoretical analysis, presented the following expression,

(4) $$\alpha =12.6\rho \frac{Z^{0.17}}{E_{max}^{1.28}}.$$

**Figure 1:**
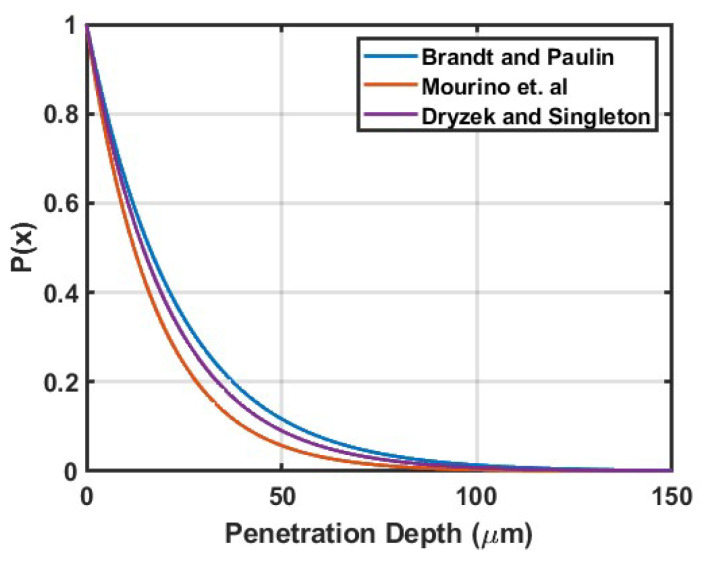
Probability functions for the implantation depths of positrons from a ^22^Na source are plotted for the three linear absorption coefficients referenced from previous literature [25,26,28].

The implantation profiles using the linear absorption coefficients above are shown in Figure 1. There are differences in calculated linear absorption coefficients determined from the three different expressions. It should be noted that the error associated with the value calculated through the Drzyek and Singleton method is about 30%, which was much higher than many of the other materials analyzed, and thus some variation in the profiles from various works should be expected. Even with the variation, it is clear that the vast majority of positrons are expected to be stopped within about 100 μm of the molybdenum surface. This illustrates the fact that the positrons are sampling a three-dimensional space within the molybdenum, dependent on the energy of the positron and the distribution of the radioactive material within the active area of the source.

The most common method for conducting positron measurements involves sandwiching the source between two, nominally identical, samples of the material under test. The deposited source covers an area within the roughly 57 mm^2^ active area which is completely covered on both sides by the sandwich configuration of the specimens under test. While encapsulating the source in foil prevents contamination of the laboratory or samples, annihilations within the ^22^Na crystallites and titanium foil can complicate lifetime spectra. The collected positron lifetime spectra will thus include components from the material under study, two components from the source itself, and potentially a longer (∼1 ns) component that can be assigned to surface interactions. Understanding the contributions of the source to the collected lifetime spectra is critical to the successful interpretation of the data.

Generally, source contributions are determined experimentally by measuring the lifetime spectra of defect-free materials. In this work, two polished 12 × 12 × 1 mm single crystal copper substrates (Stanford Advanced Materials, Lake Forest, CA, USA), were used to analyze the source contribution. Experimentally, positrons in defect-free copper have a lifetime of 120 ps [29]. The spectrum, containing more than 10${}^{7}$ counts, was deconvoluted using the PALSfit3 version 3.237 software package [30]. The deconvolution of the lifetime spectrum yielded the three lifetimes and intensities shown in Table 1. The first-lifetime component represents the copper while the second and third-lifetime components were attributed to the annihilations occurring in the source. The expected experimental lifetimes of annealed and defected titanium are between 150 and 190 ps depending on the defected state of the metal [31]. The increase in the lifetime component to 282.9 ps is due to a convolution of the annihilation signals from the foil and NaCl crystallites, which have a lifetime of around 430 ps. The 745.1 ps lifetime can be attributed to interactions with the surfaces of the source [32].

Only a small number of reports have been published focusing on the effect of source contributions to positron lifetime measurements and the majority of these only assess Kapton as the foil material [32,33,34,35]. Staab et al. [36] addressed source corrections in aluminum-encapsulated sources while McGuire and Keeble included nickel foil as an additional material to both Kapton and aluminum. In these studies, a 10 μCi source was deposited on aluminum, Kapton, and nickel source foils with thicknesses of 2 μm, 7 μm, and 5 μm, respectively. Annihilation in the foils was determined to be about 5%, 16%, and 38%, respectively, when testing molybdenum samples. The intensity of the source contributions is a function of the atomic number of the material under test and the thickness and electron density of the encapsulating material. Based on the previously reported source contribution numbers, the roughly 20% contribution from the titanium foil used in our experiment is reasonable.

The source correction was applied to all lifetime spectra containing more than 10^7^ total counts. Spectra were analyzed using the POSITRONFIT module within PALSfit3 [30]. An average background was subtracted from all spectra and a fitting routine was developed to ensure all analyses were consistent. PALSfit3 calculates a goodness-of-fit value, the reduced $\chi^{2}$ parameter, for all fits. The reduced $\chi^{2}$ parameter should be minimized with a value as close to unity as possible. This number is not consistently reported in the literature for PALS studies; however, it appears that a value of less than 1.2 is generally considered reasonable [32,37].

**Figure 2:**
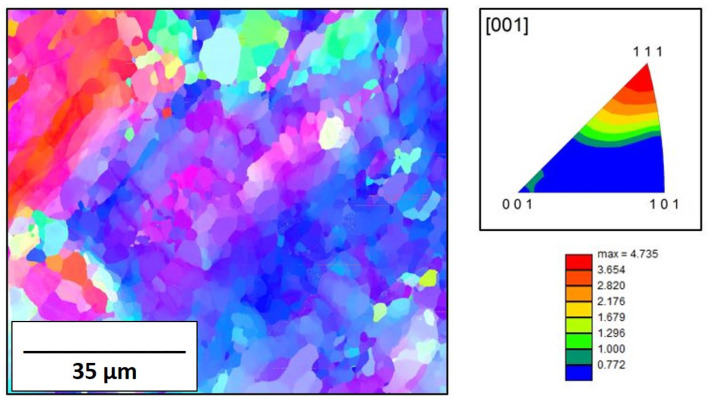
EBSD inverse pole figure (IPF) map for traditionally manufactured molybdenum with determined texture intensity.

**Figure 3:**
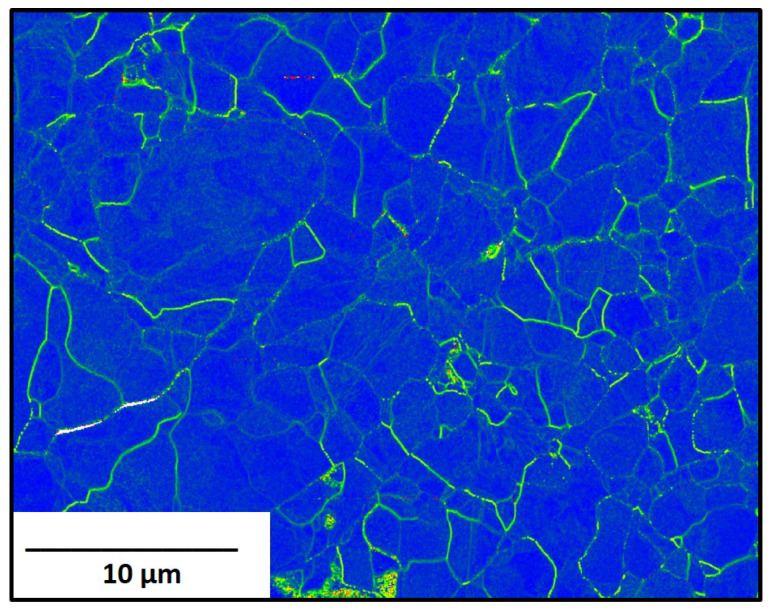
KAM map for traditionally manufactured molybdenum.

A molybdenum standard, manufactured per ASTM-B-387-18 [38] underwent initial EBSD and PALS measurements. The EBSD map for wrought molybdenum is shown in Figure 2 and has a dense, equiaxed, microstructure with a strong <111> fiber texture. The average grain diameters were determined to be 4.66 ± 0.28 μm. The kernel average misorientation (KAM) map, Figure 3, shows the differences in strain found within the grains and the grain boundaries (highlighted by the green color). By the standard, ASTM-B-387-18, the molybdenum products are to be delivered in a wrought and stress-relieved state.

Analysis of the lifetime spectra for the molybdenum standard resulted in a single lifetime component of 136.1 ± 0.1 ps. The expected lifetime of bulk (defect-free) molybdenum is around 120 ps [39,40]; however, untreated molybdenum has been shown to have a first component lifetime of around 140 ps [2]. This lifetime between 130 and 140 ps, which is higher than the bulk lifetime, but lower than the monovacancy lifetime has been attributed to annihilations at dislocations and their intersections [3]. Staab et al. [41] showed that Monte Carlo simulations indicate that a measurable percentage (1–3%) of annihilations occur at the grain boundaries for materials with grain sizes less than 15 μm; however, the lifetime spectrum for the molybdenum standard could not be reasonably fit with two lifetime parameters so there is no indication of annihilations at grain boundaries present in our analysis.

## 3. Results

Specimens of pure molybdenum and Mo-0.1SiC were prepared across a range of four scan speeds: 100, 200, 400, and 800 mm/s. This work will report the results of EBSD texture and grain boundary analysis along with PALS measurements. The results of mechanical testing, chemical composition, and optically determined density analyses, and scanning and transmission electron microscopy coupled with energy dispersive X-ray spectroscopy were presented in our previous paper [21].

### 3.1. EBSD Analysis

Microstructural characterization was conducted by collecting EBSD maps on each of the LPBF specimens. Three maps were taken of each specimen at view fields that were as large as possible. Analysis of the specimens were conducted using the EDAX OIM Analysis software. Inverse pole figure (IPF) maps were collected and used to determine the average grain diameters and crystallographic texture. KAM maps provided pixel-by-pixel analysis of orientation at a sub-grain resolution. Thresholds were placed on misorientation to remove larger misorientation associated with grain boundaries. Grain orientation spread (GOS) graphs provide another method of measuring the misorientation within a grain by averaging over the entire grain. Grain boundary analysis was conducted to determine the fraction of high-angle grain boundaries (HAGB). Clusters of vacancies, which can occur at HAGB, and grain boundaries themselves act as positron trapping sites due to their localized negative charge so knowledge of these microstructural features is important for analyzing the positron lifetime data.

**Figure 4:**
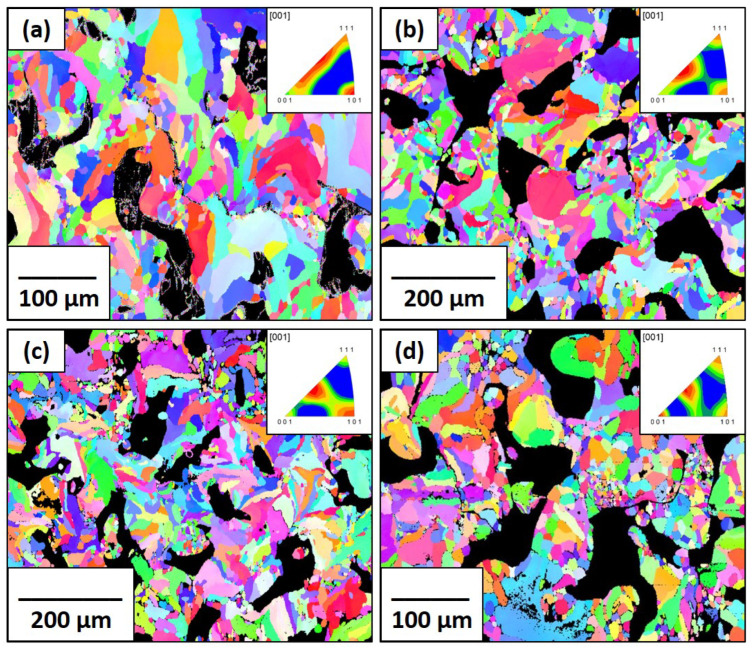
EBSD IPF maps and corresponding IPFs for the pure molybdenum specimens in the observation direction for all four scan speeds: (**a**) 100 mm/s; (**b**) 200 mm/s; (**c**) 400 mm/s; (**d**) 800 mm/s.

**Figure 5:**
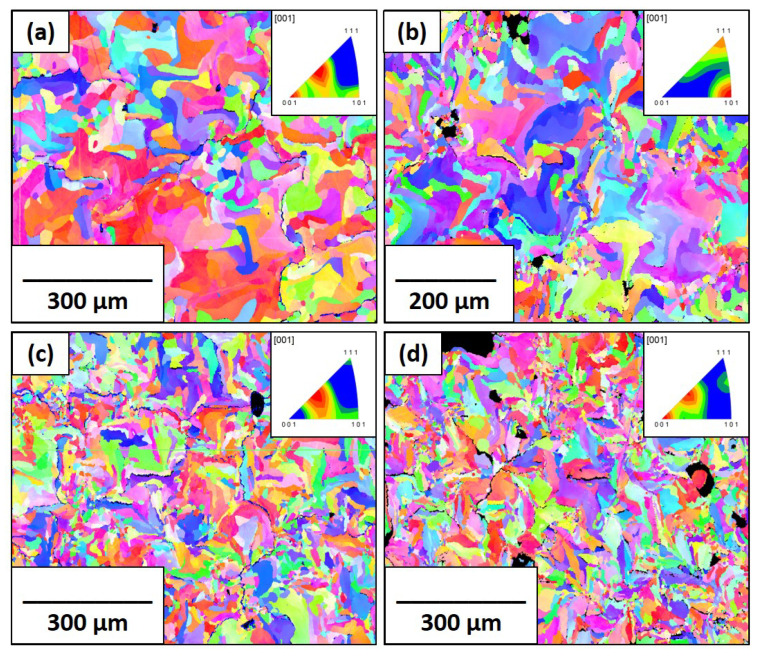
EBSD IPF maps and corresponding IPFs for the Mo-0.1SiC specimens in the observation direction for all four scan speeds: (**a**) 100 mm/s; (**b**) 200 mm/s; (**c**) 400 mm/s; (**d**) 800 mm/s.

The EBSD IPF maps of the pure molybdenum and Mo-0.1SiC specimens with crystallographic textures are shown in Figure 4 and Figure 5, respectively. As mentioned in our previous work [21], a significant amount of porosity plagued the prints of pure molybdenum and this can easily be seen even at the scales shown. Porosity was significantly reduced in the Mo-0.1SiC specimens. There is not a readily apparent effect of scan speed on the fiber texture in the transverse direction for either set of samples, as all textures appear random. This is in line with results obtained by Higashi et al. [42] who reported a loss of preferred crystallographic texture in samples with an abundance of lack of fusion pores.

The average grain diameters determined from the IPF maps are shown in Table 2. The grain sizes of the pure molybdenum specimens at the 100, 200, and 400 mm/s scan speeds are all very close, implying the increase in scan speed, and thus energy input did not appreciably alter the grain growth. There is a significant difference in the grain sizes between 400 and 800 mm/s corresponding to a larger difference in the melt pool temperature profile. This is mirrored in the Mo-0.1SiC specimens, where the expected trend is inversely related to scan speed after the 200 mm/s scan speed. From our previous research, it is known that, at the same processing parameters, the Mo-0.1SiC system experiences considerably more energy input than the pure molybdenum, and the differences with scan speed are more emphasized [21]. The differences between the grain sizes of pure molybdenum and Mo-0.1SiC demonstrate that the effect of the silicon carbide additions is more influential at lower scan speeds.

**Figure 6:**
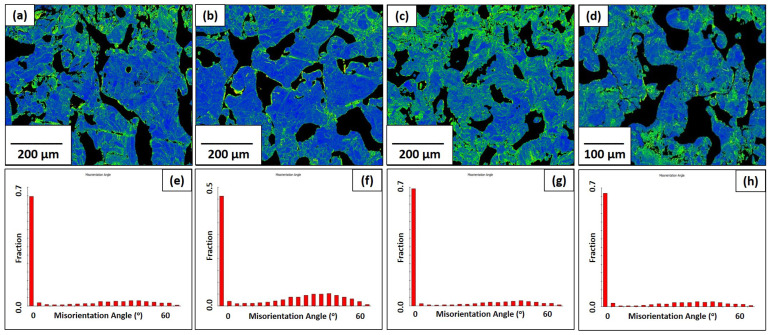
KAM maps for the pure molybdenum specimens in the observation direction at all four scan speeds: (**a**) 100 mm/s; (**b**) 200 mm/s; (**c**) 400 mm/s; (**d**) 800 mm/s. Grain orientation spread graphs for the pure molybdenum specimens in the observation direction at all four scan speeds: (**e**) 100 mm/s; (**f**) 200 mm/s; (**g**) 400 mm/s; (**h**) 800 mm/s.

**Figure 7:**
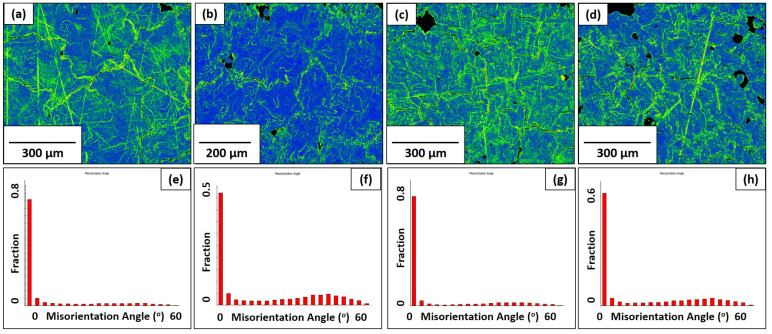
KAM maps for the Mo-0.1SiC specimens in the observation direction at all four scan speeds: (**a**) 100 mm/s; (**b**) 200 mm/s; (**c**) 400 mm/s; (**d**) 800 mm/s. Grain orientation spread graphs for the Mo-0.1SiC specimens in the observation direction at all four scan speeds: (**e**) 100 mm/s; (**f**) 200 mm/s; (**g**) 400 mm/s; (**h**) 800 mm/s.

Both the KAM maps and GOS graphs are shown for the pure molybdenum and Mo-0.1SiC specimens in Figure 6 and Figure 7. The traditionally manufactured molybdenum exhibits less kernel misorientation than what is seen in the LBPF specimens, which indicates the expected reduced residual stress from the stress-relieving treatment. The pattern of average GOS values for the pure molybdenum specimens mirrors the grain sizes for the scan speeds of 100, 200, and 400 mm/s. Limited grain growth is coupled with limited recrystallization and reduced lattice strain. The average GOS value found in the 800 mm/s specimen, is greater than anticipated due to the grain structure of poorly melted powder particles. Figure 4d shows these poorly restructured grains. The average GOS value of the Mo-0.1SiC specimen at 200 mm/s is unexpectedly increased compared to the other scan speeds.

HAGBs are generally considered to be those with a misorientation angle that exceeds 15° [43]. The fractions of HAGB found in the LPBF specimens are shown in Table 3. As with the other analyses, the pure molybdenum specimens do not display a clearly identifiable trend with the print parameters. As the grain sizes decrease, HAGBs are expected to become more prevalent [44]; however, this is not observed in pure molybdenum. Other than at the 200 mm/s scan speed, the fractions of HAGB in the Mo-0.1SiC specimens follow this trend.

### 3.2. Positron Lifetime Measurements

All positron lifetime measurements collected for the molybdenum and Mo-0.1SiC specimens yielded complex lifetime spectra that required at least two components to provide optimal fits. Initial guesses of lifetime parameters were chosen as 115 and 170 ps, representing the characteristics of experimental lifetimes of the bulk and defect-free regions in molybdenum [29]. The results of the initial deconvolution of the PALS spectra are shown below in Table 4 and Table 5. After the removal of a constant background and determined source contribution, the initial analysis employed two unconstrained lifetime fitting parameters.

The long lifetime component found in the pure molybdenum specimens is likely due to a contribution of annihilations associated with the formation of positronium in regions of open volume (such as the porosity present in these samples) or along free surfaces [18]. There was a long lifetime component removed due to the source contribution analysis; however, this does not fully account for the positron lifetimes found in the pure molybdenum LPBF samples. The copper and molybdenum standards measured as part of this analysis do not exhibit the same open porosity where positronium may be likely to form and therefore this component was not corrected for with the initial source contribution analysis. The formation and annihilation of positronium are not considered a bulk process in this case and will therefore be removed by constraining lifetime parameters during subsequent analyses.

Generally, it appears that the Mo-0.1SiC specimens are more defected as the $\tau_{1}$ component has an intensity significantly less than that of the $\tau_{1}$ component in the molybdenum specimens. Additionally, I_2_, corresponding to a second-lifetime component, usually associated with defects [18], is more prevalent.

In a defect-free solid, positron annihilations are characterized by a single lifetime. When trapping sites are available, differences in annihilation rates due to variations in electron density result in a range of lifetimes corresponding specifically to the type of trapping site where they occur. Previous PAS studies of molybdenum have yielded the lifetimes gathered in Table 6. Included in this table is the positron lifetime for silicon dioxide (SiO_2_) due to previous identification of silicon and oxygen-containing secondary-phase nanoparticles in the Mo-0.1SiC specimens [21].

The range of lifetimes associated with microvoids is due to the lifetime of positrons in a condensed material correlating with the size of the void in which they annihilate. The larger the void, the longer the associated lifetime [20]. The 350 to 450 ps range corresponds to microvoids that contain 5–15 vacancies.

The lifetimes determined in the unconstrained analysis do not necessarily match any of the known lifetimes of positrons found in molybdenum. It is likely that the unconstrained analysis yielded lifetimes that remain convolutions of multiple lifetimes. Attempting an unconstrained analysis with additional lifetime parameters was unsuccessful. Due to this, an iterative approach that involved constraining different combinations of the lifetimes presented in Table 6 was adopted. Due to the suspected positronium formation, a long lifetime of 1 ns was constrained. In addition to this, the constrained parameters chosen for the molybdenum specimens were 115, 135, and 430 ps corresponding to bulk, dislocations, and microvoid trapping sites, respectively. The $\tau_{1}$ component in the unconstrained fit for Mo-0.1SiC was around the dislocation lifetime and through the iterative process it was clear that the shorter lifetime found in the molybdenum specimens was not present. With the presence of the silicon-oxide nanoparticles in mind, the constrained parameters for the Mo-0.1SiC specimens were the 135, 261, and 430 ps corresponding to the dislocation, SiO_2_, and microvoids, respectively. It should be noted that while the reduced-$\chi^{2}$ values for certain spectra increased, the constrained fits are consistent with those for the unconstrained fits and are less than 1.2.

**Figure 8:**
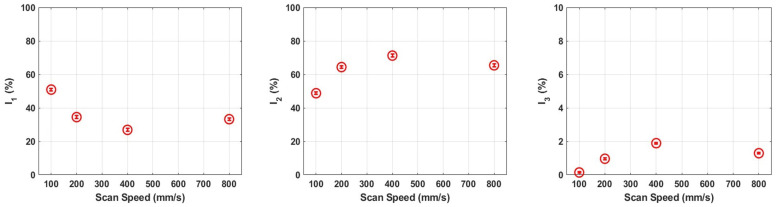
Constrained fitting for LPBF molybdenum. The I_1_, I_2_, and I_3_ correspond to the three fixed lifetime components: 115, 135, and 430 ps, respectively.

**Figure 9:**
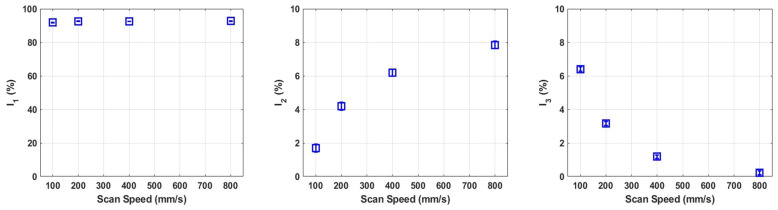
Constrained fitting for LPBF Mo-0.1SiC. The intensities correspond to the three fixed lifetime components: 135, 261, and 430 ps.

Figure 8 and Figure 9 show the intensities of the fixed components as a result of this analysis in the pure molybdenum specimens and Mo-0.1SiC specimens, respectively. The 1 ns lifetime associated with positronium formation is omitted as it is not considered a bulk effect in condensed metals. The intensity of this lifetime was less than 1% for all spectra.

After the first scan speed at 100 mm/s, the intensity of $\tau_{1}$, the defect-free component, drops between 15–20%. Correspondingly the intensity of $\tau_{2}$, which corresponds to jogs or intersections of dislocations increases by roughly the same amount. In the third component, $\tau_{3}$, the intensity only increases over 1.5% for the 400 and 800 mm/s scan speeds. This, along with the unconstrained analysis, suggests that the annihilation rate in microvoids only begins to compete with the annihilation rates in defect-free regions and dislocations in the pure molybdenum specimens at faster scan speeds.

It was clear from the unconstrained analysis that the shorter lifetime exhibited in the molybdenum specimens was not present in the Mo-0.1SiC, and indeed it appears that the 135 ps lifetime is predominant at a stable intensity of about 92% across the four scan speeds. In this fitting procedure, $\tau_{2}$ is 261 ps and represents the presence of SiO_2_. The intensity of $\tau_{2}$ increases by about 6.5% from the print conducted at 100 mm/s to that at 800 mm/s. The microvoid component, $\tau_{3}$, has an intensity at the 100 mm/s scan speed that is significantly higher than any found in the pure molybdenum specimens. This decreases monotonically with increasing scan speed and is essentially nonexistent in the specimen printed at 800 mm/s.

## 4. Discussion

Our earlier work [21] discussed the implications of the nano-sized additions of silicon carbide to LPBF molybdenum in detail, and the results of the EBSD analysis in this paper are presented to provide a substantiated foundation from which to interpret the positron lifetime spectra. As previously mentioned, the positron lifetime measurements for all of the LPBF specimens yielded complex lifetime spectra. The occurrence of multiple lifetime components is a result of numerous trapping processes present in the material. The microstructure of LPBF materials is often complicated by the conditions at which the materials are consolidated. While microscopy techniques such as SEM, TEM, EBSD, and EDS, provide very useful information, they are inherently destructive and require preparation that can, in some cases, be quite significant. PAS techniques offer the potential of bulk, defect analysis in a non-destructive manner, but the assignment of lifetimes to specific trapping sites can be complicated by influences from both the grain boundaries and defects within the grains due to the finite timing resolution of radiation detection equipment. The goal of this discussion is to use the microstructural information provided by the EBSD analysis to examine the possible trapping sites within LPBF molybdenum and Mo-0.1SiC and establish whether or not it is likely that these are contributing to the annihilation characteristics.

### 4.1. Grain Boundaries

Positrons can reach depths of up to 100–150 μm in molybdenum using the ^22^Na source. Once implanted, thermalization occurs rapidly, within about a few picoseconds [49]. Once thermalized, the positron will diffuse through the lattice in a random-walk manner. In molybdenum, the diffusion length is less than 100 nm and is dependent on the defect concentration in the lattice, with positrons in more annealed samples having an increased diffusion length [50]. The probability of annihilation at a specific trapping site is proportional to the probability of diffusing to that trapping site and due to grain size (see Table 2) and diffusion length difference, which are at least three orders of magnitude larger, this probability is very small [37]. The lifetime of positrons would be expected to be in the 350–450 ps ($\tau_{3}$ component) range associated with microvoids. The average grain diameter for the traditionally manufactured molybdenum was much smaller than for the LPBF specimens; however, there was no indication of a longer lifetime component that could be associated with grain boundaries as only a single lifetime component of 136.1 ps was identified. In the Mo-0.1SiC specimens, the $\tau_{3}$ intensity decreases with increasing scan speed. If this intensity correlated with grain size, we would expect to see an increase in the intensity due to the presence of more HAGB at higher scan speeds. The $\tau_{3}$ intensity trend mirrors the trend of the HAGB for the pure molybdenum specimens within the error of the calculated. It is well known that oxygen segregates to the grain boundaries in LPBF molybdenum [9]. Our previous research indicates the pure molybdenum specimens contain a significantly higher fraction of oxygen than the Mo-0.1SiC specimens [21]. Positrons have a high affinity for oxygen and it is possible a stronger correlation to the grain boundaries is occurring in the pure LPBF molybdenum as opposed to the molybdenum standard and Mo-0.1SiC specimens; however the contribution to the total annihilation rate is minimal. It is clear that the grain boundaries are not influencing the positron lifetime spectra in a meaningful way which indicates the majority of trapping sites are located within the grains themselves. With this conclusion, the analysis of the positron lifetimes will continue with the understanding that it is sampling the sub-grain nano-structure as opposed to the grain boundary microstructure.

### 4.2. Defect-Free Bulk Material

In well-annealed materials, the most common channel for positron annihilation is the bulk region where the crystal lattice exhibits no defects. This lifetime is generally very low for all metals and, as mentioned previously, is 115 ps in molybdenum. The two-component unconstrained analysis indicated that this lifetime was present in the pure molybdenum but not the Mo-0.1SiC specimens. Figure 8 shows that the 115 ps ($\tau_{1}$) component for the pure molybdenum specimens decreases from ∼51% at the 100 mm/s to ∼27% at 400 mm/s before increasing back to ∼33% at 800 mm/s. The GOS graph and EBSD image for the 800 mm/s scan speed imply that some of the grain structure is retained from the original powder particles due to never fully melting, accounting for this PALS result. The laser scan speed influences the solidification rates of the melt pool. At lower scan speeds the cooling rate that the melt pool experiences is slower which provides more time and energy for recovery and recrystallization to occur within the grains. Due to this, it is expected that the defect-free lifetime would be more intense in the specimens at lower scan speeds. The average grain sizes do not clearly indicate a difference in thermal profiles between the 100, 200, and 400 mm/s as there is no significant difference in crystal growth. The $\tau_{1}$ intensities strongly indicate a reduction in thermal energy is occurring. It is supposed that this is indicating in situ annealing at the lower scan speed.

### 4.3. Dislocations

Dislocations are linear defects in crystalline materials that influence many macroscopic properties. They can be created by the external loading or unloading as well as the agglomeration of self-interstitial atoms [51]. The lifetime associated with dislocations (∼135–140 ps) was noted in all the samples measured to include the traditionally manufactured molybdenum. It is very likely that LPBF and the rolling or extrusion process (for the molybdenum standard) resulted in the formation of dislocations so we can say with confidence that these are acting as the trapping sites associated with this lifetime. The production of the molybdenum shown in Figure 2 involved a mechanical process where strain was introduced to the microstructure generating dislocations. The molybdenum was stress-relieved to the point where distortions would not occur; however, there is no indication from the positron data that the temperature was high enough during this stress-relief period to cause the dislocation density to decrease. Standard stress-relief temperatures for molybdenum are between 870 and 980 °C [52]. Positron studies of deformed molybdenum have shown decreased dislocation density at 500 °C after one hour of annealing [3]. It is possible that the mechanical processes used to prepare a section of the molybdenum bar for positron analysis resulted in an increase in dislocation density. The positron spectra for both sets of LPBF specimens indicate the presence of dislocations (135 ps lifetime component). The intensity of the 135 ps lifetime component, in the pure molybdenum specimens, increases by ∼20% across the 100, 200, and 400 mm/s scan speeds. At the 100 m/s scan speed, the defect-free component is more prevalent, but the dislocation component is more prevalent at faster speeds. This is likely caused by the rapid solidification of the melt pool, which results in smaller, less recrystallized grains. The intensity falls at 800 mm/s due to, again, incomplete melting of the powder particles and a higher prevalence of defect-free regions. Unlike the pure molybdenum samples, the dislocation lifetime component is the predominant component in the Mo-0.1SiC specimens at a consistent intensity of ∼92% for all four scan speeds. Dislocations form around nanoparticles in a metal matrix, and these dislocations can become intersected and tangled [53]. The large intensity associated with the dislocation lifetime is due to the presence of silicon and oxygen-containing nanoparticles distributed within the molybdenum grains. The annihilation of positrons in these dislocations is favored over the bulk metal due to reduced atomic density. The EBSD analysis does not provide evidence of this vast disparity in dislocation behavior as the influence of the nanoparticles in the grains is not captured by any of the analyses.

### 4.4. Secondary Phases

Two types of secondary-phase nanoparticles were found in the LPBF specimens. Evidence of molybdenum oxides was revealed through SEM on the fracture surfaces of both the pure molybdenum and Mo-0.1SiC specimens while silicon oxide-containing nanoparticles were found well distributed throughout the grains of the Mo-0.1SiC specimens by TEM [21]. The solubility of oxygen in molybdenum is quite low and the segregation of the oxides formed during printing occurs at the grain boundaries [54]. The influence of the grain boundaries has been shown to be minimal, thus the presence of a lifetime corresponding to annihilations in molybdenum oxides is unlikely to be found with any significant intensity. Chemical composition analysis showed that the oxygen content was significantly higher in the pure molybdenum specimens than in the Mo-0.1SiC. The amount of oxygen increased monotonically with scan speed in the Mo-0.1SiC specimens [21]. It was clear that the increased melt pool temperature at lower scan speeds drove the reduction in oxygen; however, it was noted that the silicon sequestered an amount of oxygen into nanoparticles. As positrons diffuse through the crystal lattice, they are repelled by the surrounding atoms so any regions of decreased atomic density or increased electronic density are attractive trapping sites. Therefore, it is likely that positrons will diffuse to, and annihilate in, the silicon oxide nanoparticles. The lifetime for α-quartz, estimated to be around 261 ps, was fixed in the Mo-0.1SiC positron spectra and, as Figure 9 shows, the intensity increases monotonically with scan speed similarly to the increase shown for the oxygen content. This provides evidence that some of the additional oxygen that was found in the Mo-0.1SiC at the higher scan speeds was captured by the silicon and sequestered. It is well known that nanoparticles can pin grain boundaries in materials, thus increasing the hardness. The increasing intensity of the 261 ps lifetime can be correlated to the hardness measurements conducted on these specimens by Ellsworth et al. [21], as both indicate an increasing abundance of secondary-phase nanoparticles. This information was not readily determined through the use of SEM and TEM, further highlighting the role that bulk measurement tools such as PAS techniques can fill for AM materials.

### 4.5. Voids

The lifetime for nano-voids was fixed at 430 ps for both sets of LPBF specimens as they were expected to be present in HAGBs. As mentioned previously, HAGBs become more prevalent as grain sizes decrease. This trend was observed in the Mo-0.1SiC specimens (Table 3), but the intensity of the 430 ps component (Figure 9) decreased. This indicates that the 430 ps lifetime component corresponds to voids not present at HAGBs, but more likely within the grains themselves. The intensity of this component in the Mo-0.1SiC specimens is inversely related to the component associated with SiO_2_. As the energy deposited into the powder system increases, so too does powder vaporization. Vaporization of the metal powder can lead to melt pool instability, key-hole pores, porosity, and lack-of-fusion defects [55]. Molybdenum has a tendency to oxidize under LPBF conditions, first to molybdenum dioxide (MoO_2_) and then further to molybdenum trioixde (MoO_3_). MoO_3_ exhibits extensive volatility and vaporizes readily at the high temperatures seen during LPBF [56]. At higher scan speeds, the overall energy supplied to the powder system is lower resulting in less vaporization and more oxygen present in the consolidated component. While the 430 ps component corresponds to voids that are 5–15 vacancies in size which is much smaller than the standard size of key-hole pores, which are generally on the order of dozens of microns [57], it is likely that these voids present in the grain are due to the evaporation of trapped gases but are not big enough to be considered key-hole pores.

## 5. Conclusions

The comparison of the results of the EBSD analyses and the PALS measurements has yielded the following conclusions:

1. Grain boundary evolution during the LPBF process was observable through IPF plots and KAM maps; however, their influence on the positron annihilation characteristics was insignificant, indicating the positrons were sampling sub-grain nano-scale features.
2. PALS measurements highlight the presence of silicon oxide nanoparticles and the greatly increased dislocation density in the Mo-0.1SiC, a microstructural feature that was not readily observable from the EBSD analysis.
3. PALS provides evidence that the quantity of silicon oxide nanoparticles in the consolidated material increases at higher laser scan speeds, information that was similarly unobtainable from other analyses.
4. Nano-voids are present in the Mo-0.1SiC specimens as a result of metal vaporization; however, the subsurface, bulk nature of these pores does not easily allow for non-destructive analysis through traditional electron microscopy techniques.

The comparison of the positron lifetimes with the EBSD results indicates that the PALS technique is sensitive to sub-grain, nano-scale features as opposed to the larger grain boundaries at the microstructural scale. These features are not fully observed by traditional SEM analysis. The investigation of pure molybdenum and Mo-0.1SiC specimens by PALS measurements have proven the usefulness of PAS techniques to the study of AM materials by elucidating nanoparticle and atomic level defects.

## Figures and Tables

**Table 1:** Lifetime and intensity parameters that provide the best fit to the single crystal copper lifetime spectrum. The reduced-$\chi^{2}$ parameter for this fit was 1.052.

$\tau_{1}$ (ps)	I_1_ (%)	$\tau_{2}$ (ps)	I_2_ (%)	$\tau_{3}$ (ps)	I_3_ (%)
121.7 ± 1.2	77.15 ± 0.73	282.9 ± 5.1	20.77 ± 0.61	745.1 ± 20.8	2.08 ± 0.14

**Table 2:** Average grain diameters (μm) for the pure molybdenum and Mo-0.1SiC specimens corresponding to Figure 4 and Figure 5, respectively.

Scan Speed	100 (mm/s)	200 (mm/s)	400 (mm/s)	800 (mm/s)
**Molybdenum**	33.63 ± 4.34	27.63 ± 2.54	30.07 ± 3.10	22.91 ± 1.69
**Mo-0.1SiC**	47.94 ± 9.74	42.44 ± 5.89	26.32 ± 0.69	24.34 ± 1.05

**Table 3:** Fraction of boundaries with a rotation angle greater than 15${}^{\circ }$ for pure molybdenum and Mo-0.1SiC specimens.

Scan Speed	100 (mm/s)	200 (mm/s)	400 (mm/s)	800 (mm/s)
**Pure molybdenum**	0.33 ± 0.07	0.51 ± 0.03	0.45 ± 0.06	0.33 ± 0.03
**Mo-0.1SiC**	0.22 ± 0.04	0.44 ± 0.03	0.27 ± 0.08	0.40 ± 0.01

**Table 4:** Results of deconvolution using two unconstrained lifetime fitting parameters for LPBF molybdenum with reduced $\chi^{2}$ values.

Scan Speed (mm/s)	$\tau_{1}$ (ps)	$\tau_{2}$ (ps)	I_1_ (%)	I_2_ (%)	$\chi^{2}$
100	125.2 ± 0.1	751.5 ± 32.6	99.66 ± 0.02	0.34 ± 0.02	1.068
200	128.8 ± 0.2	534.1 ± 13.8	99.07 ± 0.04	0.93 ± 0.04	1.083
400	129.7 ± 0.2	414.8 ± 8.30	98.02 ± 0.09	1.97 ± 0.09	1.052
800	128.7 ± 0.2	464.4 ± 11.1	98.69 ± 0.07	1.31 ± 0.07	1.000

**Table 5:** Results of deconvolution using two unconstrained lifetime fitting parameters for LPBF Mo-0.1SiC with reduced $\chi^{2}$ values.

Scan Speed (mm/s)	$\tau_{1}$ (ps)	$\tau_{2}$ (ps)	I_1_ (%)	I_2_ (%)	$\chi^{2}$
100	135.2 ± 0.3	415.0 ± 2.7	92.47 ± 0.12	7.53 ± 0.12	1.114
200	136.8 ± 0.3	384.8 ± 4.1	94.66 ± 0.15	5.34 ± 0.15	1.112
400	138.2 ± 0.3	368.9 ± 5.6	95.87 ± 0.17	4.13 ± 0.17	1.043
800	141.2 ± 0.3	409.3 ± 7.3	97.22 ± 0.12	2.78 ± 0.12	1.069

**Table 6:** Positron lifetimes for various trapping sites in molybdenum.

Trapping Site	Positron Lifetime (ps)	Reference
Single Crystal	103	Ziegler and Schaefer [45]
Bulk	115	Hyodo et al. [46]
Dislocations	135	Dryzek and Wróbel [2,3]
Monovacancy	170	Robles et al. [29]
Divacancy	∼249	Dryzek and Wróbel, Hautojärvi et al. [3,47]
SiO_2_	∼261	Kuriplach and Barbiellini [48]
Microvoids	350–450	Hautojärvi et al. [47]

## Data Availability

The data presented in this study are available on request from the corresponding author.
